# A High-Efficacy CRISPR Interference System for Gene Function Discovery in Zymomonas mobilis

**DOI:** 10.1128/AEM.01621-20

**Published:** 2020-11-10

**Authors:** Amy B. Banta, Amy L. Enright, Cheta Siletti, Jason M. Peters

**Affiliations:** a Pharmaceutical Sciences Division, School of Pharmacy, University of Wisconsin—Madison, Madison, Wisconsin, USA; b Great Lakes Bioenergy Research Center, Wisconsin Energy Institute, University of Wisconsin—Madison, Madison, Wisconsin, USA; c Department of Bacteriology, University of Wisconsin—Madison, Madison, Wisconsin, USA; d Department of Medical Microbiology and Immunology, University of Wisconsin—Madison, Madison, Wisconsin, USA; North Carolina State University

**Keywords:** CRISPR-Cas9, Mismatch-CRISPRi, Mobile-CRISPRi, bioenergy, biofuel, lignocellulosic hydrolysate, essential genes, ribosomal proteins, pyruvate decarboxylase, hopanoid biosynthesis

## Abstract

Zymomonas mobilis is a promising biofuel producer due to its high alcohol tolerance and streamlined metabolism that efficiently converts sugar to ethanol. Z. mobilis genes are poorly characterized relative to those of model bacteria, hampering our ability to rationally engineer the genome with pathways capable of converting sugars from plant hydrolysates into valuable biofuels and bioproducts. Many of the unique properties that make Z. mobilis an attractive biofuel producer are controlled by essential genes; however, these genes cannot be manipulated using traditional genetic approaches (e.g., deletion or transposon insertion) because they are required for viability. CRISPR interference (CRISPRi) is a programmable gene knockdown system that can precisely control the timing and extent of gene repression, thus enabling targeting of essential genes. Here, we establish a stable, high-efficacy CRISPRi system in Z. mobilis that is capable of perturbing all genes—including essential genes. We show that Z. mobilis CRISPRi causes either strong knockdowns (>100-fold) using single guide RNA (sgRNA) spacers that perfectly match target genes or partial knockdowns using spacers with mismatches. We demonstrate the efficacy of Z. mobilis CRISPRi by targeting essential genes that are universally conserved in bacteria, are key to the efficient metabolism of Z. mobilis, or underlie alcohol tolerance. Our Z. mobilis CRISPRi system will enable comprehensive gene function discovery, opening a path to rational design of biofuel production strains with improved yields.

**IMPORTANCE** Biofuels produced by microbial fermentation of plant feedstocks provide renewable and sustainable energy sources that have the potential to mitigate climate change and improve energy security. Engineered strains of the bacterium Z. mobilis can convert sugars extracted from plant feedstocks into next-generation biofuels like isobutanol; however, conversion by these strains remains inefficient due to key gaps in our knowledge about genes involved in metabolism and stress responses such as alcohol tolerance. Here, we develop CRISPRi as a tool to explore gene function in Z. mobilis. We characterize genes that are essential for growth, required to ferment sugar to ethanol, and involved in resistance to isobutanol. Our Z. mobilis CRISPRi system makes it straightforward to define gene function and can be applied to improve strain engineering and increase biofuel yields.

## Abstract (precis)

Biofuels produced by microbial fermentation of plant feedstocks provide renewable and sustainable energy sources that have the potential to mitigate climate change and improve energy security. Engineered strains of the bacterium Z. mobilis can convert sugars extracted from plant feedstocks into next-generation biofuels like isobutanol; however, conversion by these strains remains inefficient due to key gaps in our knowledge about genes involved in metabolism and stress responses such as alcohol tolerance. Here, we develop CRISPRi as a tool to explore gene function in Z. mobilis. We characterize genes that are essential for growth, required to ferment sugar to ethanol, and involved in resistance to isobutanol. Our Z. mobilis CRISPRi system makes it straightforward to define gene function and can be applied to improve strain engineering and increase biofuel yields.

## INTRODUCTION

Zymomonas mobilis is a Gram-negative alphaproteobacterium with superlative properties for biofuel production (1–5) but poorly characterized gene functions (6). Z. mobilis is an efficient, natural ethanologen (7, 8) capable of fermenting glucose to ethanol at 97% of the theoretical yield (7, 9) with little energy spent on biomass (8, 10). Furthermore, Z. mobilis is highly resistant to ethanol, up to 16% (vol/vol) (8). However, our ability to engineer strains of Z. mobilis that produce high yields of advanced biofuels, such as isobutanol (IBA), has been hindered by the lack of functional information for two key gene sets: metabolic and stress response/resistance genes. Genetic analysis to identify and characterize metabolic and stress response genes could allow us to engineer strains with increased flux toward IBA and away from ethanol (3, 11), as well as strains that are resistant to hydrolysate inhibitors like acetic acid and various phenolic compounds (4).

Z. mobilis has a minimalistic metabolism with little functional redundancy (12–14). Z. mobilis converts sugars to pyruvate via the Entner-Doudoroff (ED) pathway, rather than the more commonly used but less thermodynamically favorable Embden-Meyerhof-Parnas (EMP) pathway (15–18). Metabolic models based on Z. mobilis ZM4 genome sequences (12–14, 18) revealed that central metabolic pathways like EMP glycolysis and the tricarboxylic acid (TCA) cycle are missing key enzymes (e.g., 6-phosphofructokinase and 2-oxoglutarate dehydrogenase, respectively), further limiting its metabolic plasticity. Because of its streamlined metabolism, many metabolic genes are predicted to be essential for growth in Z. mobilis.

Another defining feature of Z. mobilis physiology is the production of large quantities of hopanoids (19), i.e., triterpenoid lipids that provide resistance to environmental stresses in bacteria (20–22). Hopanoids are thought to act by altering membrane fluidity and permeability, analogous to the action of cholesterol—also a triterpenoid lipid—on eukaryotic membranes (23–25). Although other bacteria make hopanoids, Z. mobilis produces them in higher quantities, with the number of hopanoids nearly matching that of phospholipids in the cell during peak production conditions (19). Hopanoids are thought to be essential to Z. mobilis, as chemical inhibition of enzymes involved in hopanoid precursor biosynthesis inhibits growth (26, 27) and transposon insertions into hopanoid biosynthesis genes result in cells with both a wild-type and transposon mutant allele (i.e., strains with both *hpn*^+^ and *hpn*::Tn alleles) (28, 29). Consistent with having an essential role in Z. mobilis physiology, hopanoid production is correlated with the ethanol content of the growth medium (30) and mutations in hopanoid biosynthesis genes increase sensitivity to ethanol (28). Whether hopanoids are required for resistance to additional stresses, such as hydrolysate toxins or alcohols other than ethanol, is unknown.

**FIG 1:**
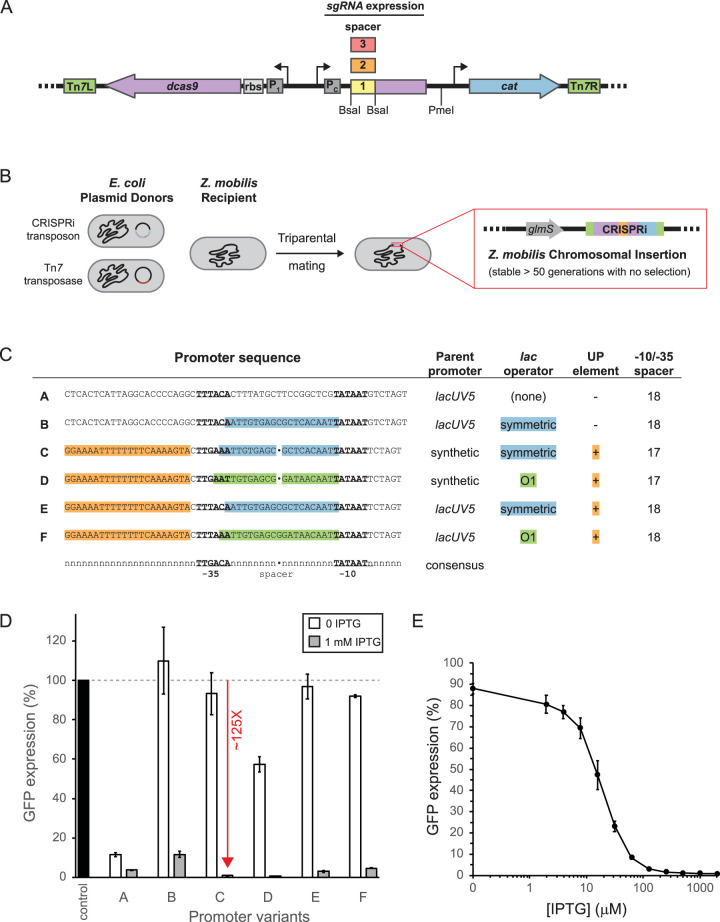
Mobile-CRISPRi system for transcriptional repression optimized for Zymomonas mobilis. (A) Modular Z. mobilis CRISPRi system encodes dCas9, sgRNA, and antibiotic resistance cassettes on a Tn*7* transposon. The promoter (P_1_) and ribosome binding site (rbs) for dCas9 and the promoter (P_C_) for the sgRNA have been optimized for Z. mobilis. DNA encoding the 20-nt variable region of the sgRNA can be cloned (individually or libraries) in between the BsaI sites. (B) CRISPRi-expressing strains are constructed by triparental mating of E. coli donor strains (one harboring the Mobile-CRISPRi plasmid and another harboring a plasmid expressing the Tn*7* transposase) with Z. mobilis. The CRISPRi expression cassette will be stably incorporated onto the Z. mobilis chromosome at the Tn*7 att* site located downstream from *glmS*. (C) Optimization of sgRNA expression. Six promoter sequences (A to F) based on either *lacUV5* or a synthetic promoter were incorporated into the CRISPRi system. Alignment is to the E. coli σ^70^ consensus promoter, with the −10 and −35 core promoter elements underlined and shown in boldface, the *lac* operator locations highlighted in green or cyan, and the UP element highlighted in orange. (D) Comparison of Z. mobilis Mobile-CRISPRi sgRNA promoter variants. A GFP expression cassette was cloned into the PmeI site, and an sgRNA targeting GFP (or a nontargeting control) was cloned into the BsaI sites. Cultures were diluted 1:1,000 and incubated in medium with 0 or 1 mM IPTG for ∼10 doublings prior to measurement of GFP expression. Expression was normalized to the results for a non GFP-expressing strain. Standard deviations of the results from 4 biological replicates are shown. (E) Expression of Z. mobilis CRISPRi system is inducible over a range of IPTG concentrations. Standard deviations are shown.

CRISPR interference (CRISPRi)—the use of programmable guide RNAs and Cas proteins (31, 32) or complexes (33, 34) lacking nuclease activity to repress transcription of target genes—is capable of probing the functions of essential genes. This is because CRISPRi knockdowns are inducible and titratable (31, 32, 35), separating the steps of strain construction and gene phenotyping. CRISPRi has been used to phenotype essential genes in multiple bacterial species (36) and define chemical-gene interactions (35, 37), cell morphology phenotypes (35, 38, 39), host genes involved in phage life cycles (40), novel gene functions (35, 38), and essential gene network architecture (35), among other purposes. To facilitate essential gene phenotyping by CRISPRi in diverse bacterial species, we recently developed “Mobile-CRISPRi”—a suite of modular CRISPRi vectors based on the extensively studied type II-A CRISPR system from Streptococcus pyogenes (i.e., Streptococcus pyogenes catalytically dead Cas9 [*Spy* dCas9]) (Fig. 1A) that are transferred by mating and site specifically integrate into the genomes of recipient bacteria (41, 42). We demonstrated integration and knockdown in species ranging from Gram-negative *Gammaproteobacteria* to Gram-positive *Firmicutes* (41), but we did not test species from *Alphaproteobacteria*. Z. mobilis contains a native type I to F CRISPR system that has been co-opted for efficient genome editing, as well as CRISPRi in a Cas3 nuclease-deficient background (34); however, CRISPRi using the native system showed low knockdown efficacy (4- to 5-fold maximum) and was not inducible as constructed, limiting its usefulness in targeting essential genes. We reasoned that importing the heterologous *Spy* dCas9 system would result in stronger knockdowns, as is seen in other species (31, 35, 41).

Here, we establish a stable and efficacious CRISPRi system for Z. mobilis based on *Spy* dCas9. We demonstrate strong (>100-fold) or partial knockdown of gene expression by using single guide RNA (sgRNA) spacers that are complementary or mismatched to target genes, respectively. We use Z. mobilis CRISPRi to demonstrate the essentiality of genes involved in metabolism and hopanoid biosynthesis. Furthermore, we show that reduced expression of specific hopanoid biosynthesis genes leads to IBA sensitivity. Our Z. mobilis CRISPRi system will enable rapid characterization of gene function, accelerating rational engineering of the genome for advanced biofuel production.

## RESULTS

### Optimization of CRISPRi for Z. mobilis.

To establish *Spy* dCas9-based CRISPRi in Z. mobilis, we first attempted to deliver a previously described Tn*7*-based Mobile-CRISPRi test construct containing the gene encoding monomeric red fluorescent protein (mRFP) and an sgRNA targeting *mRFP* (41) to wild-type Z. mobilis strain ZM4 via conjugation (Fig. 1A and B). We failed to obtain transconjugants with the wild type but succeeded at integrating Mobile-CRISPRi into the genome of a restriction-deficient derivative strain (Piyush B. Lal and Patricia J. Kiley, unpublished data), consistent with Mobile-CRISPRi vectors containing multiple predicted recognition sites for Z. mobilis restriction enzymes (43). Therefore, we used the restriction-deficient strain in all subsequent experiments (Table 1). Mobile-CRISPRi inserted into the Z. mobilis genome downstream from *glmS*, as expected (Fig. S1 in the supplemental material), and was stable over 50 generations of growth in rich medium without selection. We next used a fluorometer to measure CRISPRi knockdown of mRFP at saturating concentrations of inducer (1 mM IPTG [isopropyl-β-d-thiogalactopyranoside]), finding poor knockdown (2.4-fold) (Fig. S2), although our measurements were complicated by the weak fluorescence of mRFP in Z. mobilis.

**TABLE 1:** Strains

Strain	Description^*a*^	Reference or source
sJMP006	Escherichia coli (Keio WT strain) (BW25113) F^−^ λ^−^ *lacI*^q^ *rrnBT14* Δ*lacZWJ16 hsdR514* Δ*araBADAH33* Δ*rhaBADLD78*	62
sJMP032	Escherichia coli DH10B (cloning strain) F^−^ Δ(*ara-leu*)*7697*[Δ(*rapA′-cra′*)] Δ(*lac*)*X74*[Δ(′*yahH-mhpE*)] duplication(514341–627601)[*nmpC-gltI*] *galK16 galE15* e14^−^(*icd*^WT^ *mcrA*) ϕ80d*lacZΔ*M15 *recA1 relA1 endA1* Tn*10.10 nupG rpsL150*(Str^r^) *rph*^+^ *spoT1* Δ*(mrr-hsdRMS-mcrBC)* λ^−^ Missense(*dnaA glmS glyQ lpxK mreC murA*) Nonsense(*chiA gatZ fhuA*? *yigA ygcG*) Frameshift(*flhC mglA fruB*)	Invitrogen
sJMP146	Escherichia coli (*pir*^+^ cloning strain) (BW25141) Δ(*araD-araB*)*567* Δ*lacZ4787*(::*rrnB-3*) Δ(*phoB-phoR*)*580* λ^−^ *galU95* Δ*uidA3*::*pir*^+^ *recA1 endA9*(Δ-ins)::FRT *rph-1* Δ(*rhaD-rhaB*)*568 hsdR514*	65
sJMP412	Zymomonas mobilis ZM4 Δ*hsdS*_C_ (ZMO1933) Δ*mrr* (ZMO0028) Δ*hsdS*_R_ (pZM32_028) Δ*cas3* (ZMO0681) (strain PK15436)	Lal and Kiley, unpublished data
sJMP424	Escherichia coli (*pir*^+^ *dap*-negative mating strain) (strain WM6026) *lacI*^q^ *rrnB3* DE*lacZ4787 hsdR514* DE(*araBAD*)*567* DE(*rhaBAD*)*568 rph-1 att*-lambda::pAE12-del (*oriR6K*/*cat*::FRT5) Δ*4229*(*dapA*)::FRT(DAP^−^) Δ(*endA*)::FRT *uidA*(Δ*MluI*)::*pir*(wt) *attHK*::pJK1006::Δ1/2(Δ*oriR6K-cat*::FRT5 Δ*trfA*::FRT)	66
sJMP2032	sJMP412 with CRISPRi system from pJMP196 in Tn*7 att*, Kan^r^ (MCi-RFP-rfp)	This study
sJMP2035	sJMP412 with CRISPRi system from pJMP197 in Tn*7 att*, Kan^r^ (MCi-RFP-BsaI)	This study
sJMP2065	sJMP412 with CRISPRi system from pJMP2044 in Tn*7 att*, Cm^r^ (MCi-noGFP)	This study
sJMP2069	sJMP412 with CRISPRi system from pJMP2046 in Tn*7 att*, Cm^r^ (MCi-vA-GFP-BsaI)	This study
sJMP2073	sJMP412 with CRISPRi system from pJMP2048 in Tn*7 att*, Cm^r^ (MCi-vA-GFP-gfp)	This study
sJMP2118	sJMP412 with CRISPRi system from pJMP2093 in Tn*7 att*, Cm^r^ (MCi-vB-GFP-BsaI)	This study
sJMP2122	sJMP412 with CRISPRi system from pJMP2095 in Tn*7 att*, Cm^r^ (MCi-vB-GFP-gfp)	This study
sJMP2340	sJMP2065 with plasmid pSRK-kan (pJMP2316)	This study
sJMP2341	sJMP2065 with plasmid pSRK-kan-sgRNA (pJMP2317)	This study
sJMP2342	sJMP2065 with plasmid pSRK-kan-dCas9 (pJMP2319)	This study
sJMP2343	sJMP2069 with plasmid pSRK-kan (pJMP2316)	This study
sJMP2344	sJMP2069 with plasmid pSRK-kan-sgRNA (pJMP2317)	This study
sJMP2345	sJMP2069 with plasmid pSRK-kan-dCas9 (pJMP2319)	This study
sJMP2346	sJMP2073 with plasmid pSRK-kan (pJMP2316)	This study
sJMP2347	sJMP2073 with plasmid pSRK-kan-sgRNA (pJMP2317)	This study
sJMP2348	sJMP2073 with plasmid pSRK-kan-dCas9 (pJMP2319)	This study
sJMP2349	sJMP2118 with plasmid pSRK-kan (pJMP2316)	This study
sJMP2350	sJMP2118 with plasmid pSRK-kan-sgRNA (pJMP2317)	This study
sJMP2351	sJMP2118 with plasmid pSRK-kan-dCas9 (pJMP2319)	This study
sJMP2352	sJMP2122 with plasmid pSRK-kan (pJMP2316)	This study
sJMP2353	sJMP2122 with plasmid pSRK-kan-sgRNA (pJMP2317)	This study
sJMP2354	sJMP2122 with plasmid pSRK-kan-dCas9 (pJMP2319)	This study
sJMP2430	sJMP412 with CRISPRi system from pJMP2367 in Tn*7 att*, Cm^r^ (MCi-vC-GFP-BsaI)	This study
sJMP2433	sJMP412 with CRISPRi system from pJMP2369 in Tn*7 att*, Cm^r^ (MCi-vD-GFP-BsaI)	This study
sJMP2436	sJMP412 with CRISPRi system from pJMP2371 in Tn*7 att*, Cm^r^ (MCi-vE-GFP-BsaI)	This study
sJMP2439	sJMP412 with CRISPRi system from pJMP2373 in Tn*7 att*, Cm^r^ (MCi-vF-GFP-BsaI)	This study
sJMP2442	sJMP412 with CRISPRi system from pJMP2375 in Tn*7 att*, Cm^r^ (MCi-vC-GFP-gfp)	This study
sJMP2445	sJMP412 with CRISPRi system from pJMP2377 in Tn*7 att*, Cm^r^ (MCi-vD-GFP-gfp)	This study
sJMP2447	sJMP412 with CRISPRi system from pJMP2379 in Tn*7 att*, Cm^r^ (MCi-vE-GFP-gfp)	This study
sJMP2451	sJMP412 with CRISPRi system from pJMP2381 in Tn*7 att*, Cm^r^ (MCi-vF-GFP-gfp)	This study
sJMP2454	sJMP412 with CRISPRi system from pJMP2391 in Tn*7 att*, Cm^r^ (*hpnC* sgRNA)	This study
sJMP2456	sJMP412 with CRISPRi system from pJMP2393 in Tn*7 att*, Cm^r^ (*hpnC* sgRNA)	This study
sJMP2458	sJMP412 with CRISPRi system from pJMP2395 in Tn*7 att*, Cm^r^ (*hpnF* sgRNA)	This study
sJMP2460	sJMP412 with CRISPRi system from pJMP2397 in Tn*7 att*, Cm^r^ (*hpnF* sgRNA)	This study
sJMP2462	sJMP412 with CRISPRi system from pJMP2399 in Tn*7 att*, Cm^r^ (*hpnH* sgRNA)	This study
sJMP2463	sJMP412 with CRISPRi system from pJMP2401 in Tn*7 att*, Cm^r^ (*hpnH* sgRNA)	This study
sJMP2465	sJMP412 with CRISPRi system from pJMP2403 in Tn*7 att*, Cm^r^ (*hpnI* sgRNA)	This study
sJMP2467	sJMP412 with CRISPRi system from pJMP2405 in Tn*7 att*, Cm^r^ (*hpnI* sgRNA)	This study
sJMP2469	sJMP412 with CRISPRi system from pJMP2407 in Tn*7 att*, Cm^r^ (*shc2* sgRNA)	This study
sJMP2471	sJMP412 with CRISPRi system from pJMP2409 in Tn*7 att*, Cm^r^ (*shc2* sgRNA)	This study
sJMP2477	sJMP412 with CRISPRi system from pJMP2415 in Tn*7 att*, Cm^r^ (*rfp* sgRNA)	This study
sJMP2543	sJMP412 with CRISPRi system from pJMP2367 in Tn*7 att*, Cm^r^ (MCi-vC-GFP-BsaI)	This study
sJMP2544	sJMP412 with CRISPRi system from pJMP2375 in Tn*7 att*, Cm^r^ (MCi-vC-GFP-gfp)	This study
sJMP2545	sJMP412 with CRISPRi system from pJMP2409 in Tn*7 att*, Cm^r^ (MCi-vC-GFP-gmc1)	This study
sJMP2546	sJMP412 with CRISPRi system from pJMP2411 in Tn*7 att*, Cm^r^ (MCi-vC-GFP-gmc2)	This study
sJMP2547	sJMP412 with CRISPRi system from pJMP2413 in Tn*7 att*, Cm^r^ (MCi-vC-GFP-gmc3)	This study
sJMP2548	sJMP412 with CRISPRi system from pJMP2415 in Tn*7 att*, Cm^r^ (MCi-vC-GFP-gmc4)	This study
sJMP2549	sJMP412 with CRISPRi system from pJMP2417 in Tn*7 att*, Cm^r^ (MCi-vC-GFP-gmc5)	This study
sJMP2550	sJMP412 with CRISPRi system from pJMP2419 in Tn*7 att*, Cm^r^ (MCi-vC-GFP-gmc6)	This study
sJMP2551	sJMP412 with CRISPRi system from pJMP2421 in Tn*7 att*, Cm^r^ (MCi-vC-GFP-gmc7)	This study
sJMP2552	sJMP412 with CRISPRi system from pJMP2423 in Tn*7 att*, Cm^r^ (MCi-vC-GFP-gmc8)	This study
sJMP2553	sJMP412 with CRISPRi system from pJMP2425 in Tn*7 att*, Cm^r^ (MCi-vC-GFP-gmc9)	This study
sJMP2554	sJMP412 with CRISPRi system from pJMP2480 in Tn*7 att*, Cm^r^ (MCi-vC-noGFP-BsaI)	This study
sJMP2555	sJMP006 with CRISPRi system from pJMP2367 in Tn*7 att*, Cm^r^	This study
sJMP2556	sJMP006 with CRISPRi system from pJMP2375 in Tn*7 att*, Cm^r^	This study
sJMP2557	sJMP006 with CRISPRi system from pJMP2409 in Tn*7 att*, Cm^r^	This study
sJMP2558	sJMP006 with CRISPRi system from pJMP2411 in Tn*7 att*, Cm^r^	This study
sJMP2559	sJMP006 with CRISPRi system from pJMP2413 in Tn*7 att*, Cm^r^	This study
sJMP2560	sJMP006 with CRISPRi system from pJMP2415 in Tn*7 att*, Cm^r^	This study
sJMP2561	sJMP006 with CRISPRi system from pJMP2417 in Tn*7 att*, Cm^r^	This study
sJMP2562	sJMP006 with CRISPRi system from pJMP2419 in Tn*7 att*, Cm^r^	This study
sJMP2563	sJMP006 with CRISPRi system from pJMP2421 in Tn*7 att*, Cm^r^	This study
sJMP2564	sJMP006 with CRISPRi system from pJMP2423 in Tn*7 att*, Cm^r^	This study
sJMP2565	sJMP006 with CRISPRi system from pJMP2425 in Tn*7 att*, Cm^r^	This study
sJMP2566	sJMP006 with CRISPRi system from pJMP2480 in Tn*7 att*, Cm^r^	This study
sJMP2605	sJMP412 with CRISPRi system from pJMP2597 in Tn*7 att*, Cm^r^	This study
sJMP2606	sJMP412 with CRISPRi system from pJMP2598 in Tn*7 att*, Cm^r^	This study
sJMP2607	sJMP412 with CRISPRi system from pJMP2599 in Tn*7 att*, Cm^r^	This study
sJMP2608	sJMP412 with CRISPRi system from pJMP2600 in Tn*7 att*, Cm^r^	This study

a WT, wild type; Cm^r^, chloramphenicol resistance cassette; Amp^r^, ampicillin resistance cassette; Kan^r^, kanamycin resistance cassette; Spec^r^, spectinomycin resistance cassette; MCi, Mobile-CRISPRi; RFP, red fluorescent protein; vA to vF, sgRNA promoter variants; sfGFP, superfolder GFP.

To optimize CRISPRi function and improve knockdown detection, we took advantage of the modularity of Mobile-CRISPRi (Fig. 1A) to swap in biological parts that have been confirmed to function in Z. mobilis (44). We replaced mRFP with the gene encoding superfolder green fluorescent protein (GFP) (*sfGFP*) (45), expressed *dcas9* from a T7A1-derived promoter with a strong Z. mobilis ribosome binding site, and expressed an sgRNA targeting *sfGFP* from the *lacUV5* promoter (i.e., promoter A) (Fig. 1C). This CRISPRi system showed strong knockdown (28-fold) of sfGFP at a saturating inducer concentration, but also considerable leakiness (9-fold) without inducer (Fig. 1D, promoter A). Inserting a symmetric *lac* operator site (46) into the *lacUV5* promoter spacer (Fig. 1C, promoter B) resulted in no detectable leakiness but only modest knockdown (9-fold) (Fig. 1D), suggesting that the concentration of either dCas9 or the sgRNA was limiting for knockdown. To determine the limiting factor, we expressed either the *sfGFP* sgRNA or *dcas9* from a multicopy plasmid in the context of CRISPRi with promoter B and found that sgRNA expression was primarily limiting knockdown (Fig. S3). Because *lacUV5* has the highest confirmed activity of any promoter measured in Z. mobilis (44) and because the Z. mobilis promoter consensus sequence (47) was unknown when we began our studies, we built LacI-regulated synthetic promoters based on Escherichia coli σ^70^ consensus elements (48, 49) that we reasoned could increase sgRNA expression to a higher level than *lacUV5* (Fig. 1C, promoters C to F). All four synthetic promoters improved the knockdown properties of Z. mobilis CRISPRi, but promoter C, which features consensus UP and −10 elements with a near-consensus −35 and an ideal spacer length (Fig. 1C), provided the best combination of strong knockdown (125-fold) and negligible leakiness (∼10 to 15%) (Fig. 1D). Using CRISPRi with promoter C, we found that intermediate inducer concentrations enabled titration of knockdown activity (Fig. 1E). We conclude that Mobile-CRISPRi optimized for Z. mobilis is efficacious, inducible, and titratable.

### Mismatch-CRISPRi enables knockdown gradients in Z. mobilis.

**FIG 2:**
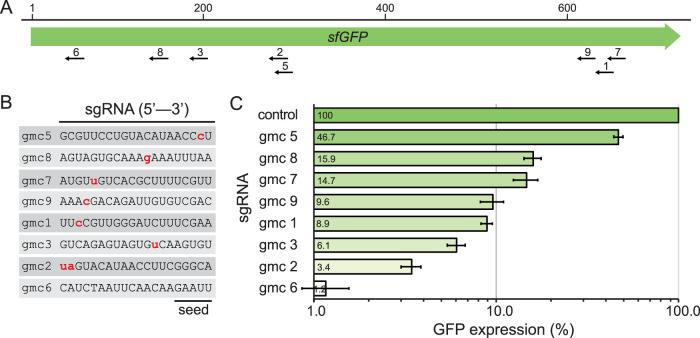
Variable levels of repression using mismatched sgRNAs in the Z. mobilis Mobile-CRISPRi system. (A) Location of sgRNA targets gmc1 to gmc9 (GFP Mismatch-CRISPRi guide RNAs) on the GFP gene. Scale bar indicates nucleotides. (B) Sequences of the GFP-targeting sgRNAs, with mismatches indicated in lowercase red. Protospacer adjacent motif (PAM)-proximal seed sequence is indicated. (C) Knockdown of GFP expression in Z. mobilis CRISPRi expression strains with mismatched sgRNAs. “Control” indicates a nontargeting sgRNA. Standard deviations are shown. IPTG (1 mM) was used for induction.

The relationship between fitness and gene expression varies by gene and is generally unknown (50, 51). This relationship is especially important to consider for essential genes, which have a fitness of zero at full knockdown but a large range of possible fitness values at intermediate levels of knockdown, depending on the function of the gene product. Excessive knockdown of essential genes results in strains that grow poorly and are difficult to phenotype. The Gross and Weissman laboratories have shown that systematically introducing mismatches between sgRNA spacers and target genes can generate knockdown gradients suitable for studying essential gene function (50–53); we call this strategy “Mismatch-CRISPRi.” Mismatch-CRISPRi functions in mammalian cells (51) and diverse model bacteria (i.e., E. coli and Bacillus subtilis [50]), although the behavior of mismatched guides is not identical in mammalian and bacterial systems (50). Hawkins and Silvis et al. took a machine learning approach to characterize the behavior of mismatched guides targeting *sfGFP* (∼1,500) in both E. coli and B. subtilis, finding that ∼50% of the mismatched-guide activity could be predicted by taking into account the mismatch position, changes in the free energy of sgRNA-DNA pairing, and %GC content; these predictions were validated by targeting essential genes with mismatched guides in both organisms (50). To test whether mismatched sgRNAs behave similarly in *Alphaproteobacteria*, we cloned a subset of the mismatched guides used by Hawkins and Silvis et al. to target *sfGFP* (50) into our Z. mobilis CRISPRi system with sgRNA promoter C (Fig. 2A and B). Using these mismatched guides, we were able to generate a knockdown gradient of *sfGFP* that spanned nearly 2 orders of magnitude and contained multiple sgRNAs that caused intermediate levels of knockdown at a saturating inducer concentration (Fig. 2C). Furthermore, we introduced our Z. mobilis Mismatch-CRISPRi vectors into E. coli, permitting a direct comparison of *sfGFP* knockdown in the two divergent species (Fig. S4). Consistent with a previous comparison between E. coli and B. subtilis (50), we found excellent agreement between *sfGFP* knockdown gradients in E. coli and Z. mobilis (*R*^2^ = 0.8). This demonstrates the broad utility of Mismatch-CRISPRi to predictably generate partial knockdowns in diverse bacteria and suggests that Z. mobilis CRISPRi may function well in multiple species.

### Z. mobilis CRISPRi targets essential genes.

**FIG 3:**
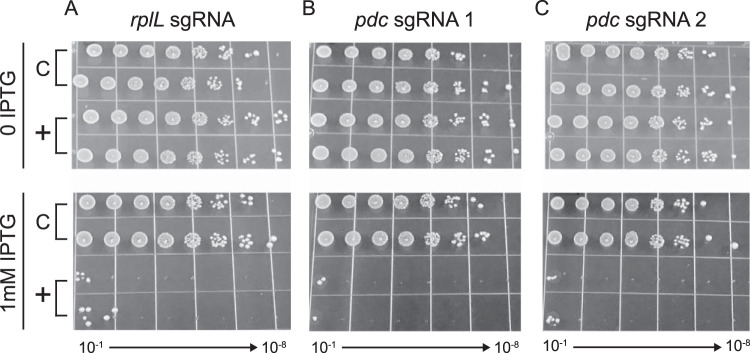
CRISPRi knockdown of endogenous essential genes. Z. mobilis strains with CRISPRi cassettes encoding sgRNAs targeting essential genes *rplL* (A) and *pdc* (B and C) were serially diluted 1:10 (10^−1^ through 10^−8^) and spotted on agar plates with either 0 or 1 mM IPTG. “C” indicates a nontargeting sgRNA (control).

To examine the efficacy of Z. mobilis CRISPRi in characterizing essential gene function, we first targeted *rplL* (ZMO0728)—an essential gene encoding the universally conserved ribosomal protein L12 (54)—as a positive control. We found a reduction greater than 6 orders of magnitude in plating efficiency for strains expressing an *rplL* sgRNA versus a control strain expressing a nontargeting sgRNA at saturating inducer (Fig. 3A), indicating substantial loss of cell viability and relatively low levels of suppressor mutations that inactivate the CRISPRi system. Based on these results, we conclude that Z. mobilis CRISPRi is effective at assessing gene essentiality and allowing observation of essential gene knockdown phenotypes.

Pyruvate decarboxylase, encoded by the *pdc* gene (ZMO1360), is a key metabolic enzyme in Z. mobilis that converts pyruvate into acetaldehyde—the penultimate step in ethanol production (55). Despite the important role of *pdc* in fermentation of sugars to ethanol, the Z. mobilis literature is conflicted about whether *pdc* is essential (5, 56) or dispensable (57, 58) under aerobic conditions. To determine the essentiality of *pdc*, we used Z. mobilis CRISPRi with promoter C and an sgRNA targeting the 5′ end of the *pdc* coding sequence. We found a loss in plating efficiency greater than 6 orders of magnitude for the *pdc* knockdown strain at a saturating inducer concentration (Fig. 3B); this result was indistinguishable from the loss of fitness observed when we targeted *rplL*, suggesting that *pdc* is essential for aerobic growth. To confirm that our result was not due to off-target effects of CRISPRi, we tested a second, nonoverlapping sgRNA targeting *pdc*, finding the same results (Fig. 3C). We conclude that *pdc* is essential for aerobic growth of Z. mobilis.

### Essentiality and IBA sensitivity phenotypes of hopanoid biosynthesis genes.

**FIG 4:**
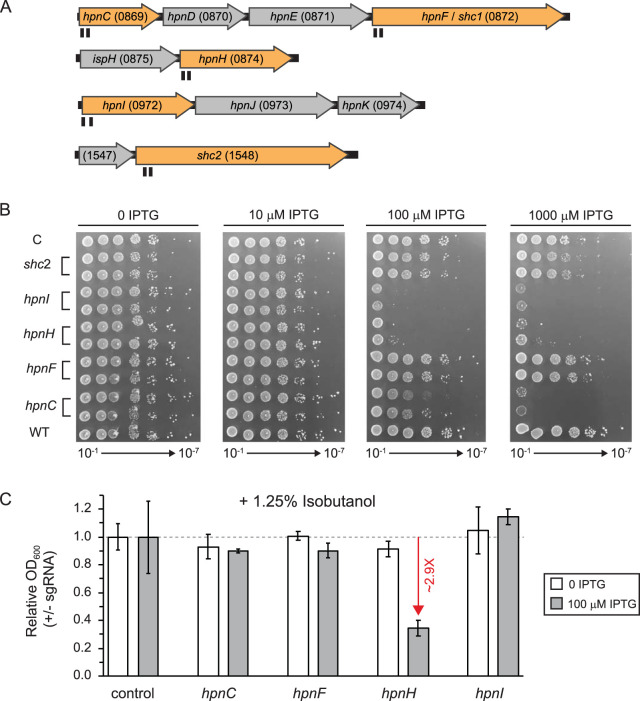
CRISPRi knockdown of hopanoid lipid synthesis-related genes. (A) Z. mobilis strains were constructed with CRISPRi cassettes encoding sgRNAs targeting genes in hopanoid synthesis operons (and a nontargeting control). The number of the ZMO locus tag is in parentheses after the gene name. Targeted genes (*hpnC*, *hpnF* [*shc1*], *hpnH*, *hpnI*, and *shc2*) are shown in orange. Target positions of sgRNAs are shown in black under the genes. (B) Strains were serially diluted 1:10 (10^−2^ through 10^−8^) and spotted on agar plates with 0, 0.01, 0.1, or 1 mM IPTG. “C” indicates a nontargeting sgRNA (control). (C) Strains were diluted 1:1,000 and grown for ∼10 doublings in liquid culture, aerobically, in the presence of 1.25% isobutanol and 0 or 0.1 mM IPTG prior to measurement of cell density (OD_600_). Growth measurements were normalized to the growth of a strain expressing a nontargeting sgRNA. Standard deviations of the results from 4 replicates are shown. Red arrow indicates fold change compared to control.

Genes encoding hopanoid biosynthesis enzymes (i.e., *hpn* and *shc* genes) (Fig. 4A and Fig. S5) are thought to be essential in Z. mobilis, based on growth cessation caused by small-molecule inhibitors of squalene-hopene cyclase (26) and the observation that strains with transposon insertions in *hpn* genes always also contain a wild-type copy of the gene (28). To further probe the essentiality of hopanoids, we targeted *hpn* and *shc* genes using Z. mobilis CRISPRi. Because CRISPRi blocks transcription of downstream genes in an operon (i.e., polarity), we chose to target the first *hpn* or *shc* gene present in each operon (Fig. 4A, orange genes). We found considerable defects in plating efficiency for strains with sgRNAs targeting *hpnC* (ZMO0869), *hpnH* (ZMO0874), and *hpnI* (ZMO0972), consistent with a requirement of hopanoid synthesis for growth (Fig. 4B). In contrast, targeting the *hpnF* (also known as *shc1* [ZMO0872]) and *shc2* (ZMO1548) genes that both encode squalene-hopene cyclase had no effect on plating efficiency, suggesting that they are functionally redundant under the conditions tested.

Classic studies of Z. mobilis physiology (27) and contemporary work using strains with both *hpn*^+^ and *hpn*::Tn alleles (28) have linked hopanoid production and ethanol concentration or resistance; however, it is unknown whether hopanoids provide resistance to advanced biofuels, such as IBA. To examine the relationship between *hpn* and *shc* genes, we first determined the concentration of IBA needed to partially inhibit growth of Z. mobilis in sealed 96-well deep-well plates. We found that the addition of 1.25% (vol/vol) IBA to rich medium inhibited Z. mobilis growth by ∼50% (Fig. S6). We then grew our CRISPRi strains targeting *hpn-shc* operon genes with a subsaturating concentration of inducer (100 μM IPTG) for a limited number of generations in the presence or absence of IBA. Under these conditions, the strain in which *hpnH* was targeted was the only knockdown tested that showed increased sensitivity to IBA, with a 2.9-fold reduction in the final optical density at 600 nm (OD_600_) at the end of the growth period relative to that of a nontargeting control sgRNA strain (Fig. 4C). HpnH performs the first enzymatic step in hopanoid side chain synthesis (Fig. S5) (59), suggesting that buildup of the core hopanoids diploptene and diplopterol may compromise IBA tolerance in Z. mobilis. We conclude that hopanoid biosynthesis operons are essential in Z. mobilis but knockdown of extended hopanoids does not alter IBA tolerance.

## DISCUSSION

The lack of genetic tools for bioenergy-relevant, nonmodel bacteria has slowed progress toward engineering efficient production strains for advanced biofuels, such as IBA. Our optimized CRISPRi system for Z. mobilis overcomes this obstacle by enabling programmable, inducible, and titratable control of overexpression of both nonessential and essential genes. Because Z. mobilis CRISPRi also functions well in the distantly related bacterium E. coli, the system as constructed may have broad utility across species. Our path to optimizing Mobile-CRISPRi for Z. mobilis revealed valuable lessons that may be generalizable across species: first, identify limiting components (either dCas9 or sgRNAs) by overexpression, and second, design strong synthetic promoters that take advantage of conserved interactions between RNA polymerase holoenzyme and DNA (48, 49) to improve portability.

CRISPRi is the ideal genetic tool to explore the unusual genetics of Z. mobilis. Because many metabolic genes are predicted to be essential (60), controlling metabolic flux may require constructing strains with partial knockdowns of essential genes. Mismatch-CRISPRi libraries are particularly well suited for empirically defining relationships between knockdown of metabolic genes (and associated changes in flux) and fitness, as strains comprising knockdown gradients of metabolic genes can be pooled and tested under a variety of growth conditions, with fitness measured by next-generation sequencing of sgRNA spacers. Furthermore, the Z. mobilis chromosome is possibly polyploid (28, 29), or at least capable of duplicating at high frequency; this can cause problems with deletion/transposon insertion analysis of essential genes or other genes that have a strong impact on fitness. For instance, a high-throughput analysis of isolated transposon insertion mutants revealed that there was no significant difference in the probability of a transposon inserting into a predicted essential versus nonessential gene (29), suggesting a polyploid chromosome and underscoring issues with interpreting insertion/deletion results in Z. mobilis. In contrast, CRISPRi is largely unaffected by polyploidy—it is capable of targeting essential genes across multiple copies of the chromosome as long as the sgRNA-dCas9 complex is expressed at high enough levels to account for additional targets.

Numerous studies have linked hopanoid production and ethanol tolerance in Z. mobilis (27, 30, 61), but whether hopanoids provide resistance to nonphysiological alcohols, such as IBA, remains unclear. Our CRISPRi results suggest that wild-type levels of extended hopanoids do not impart IBA resistance, and instead, that preventing synthesis of extended (C_35_) hopanoids by blocking *hpnH* expression causes sensitivity. The simplest explanation for these results is that the core C_30_ hopanoids, diploptene and diplopterol, accumulate in the cell and negatively impact the outer membrane. Further evaluation of this hypothesis will require careful tracking of the relationships between knockdown extent, fitness, IBA concentration, and levels of individual hopanoid species.

CRISPRi enables essential gene phenotyping, but we currently lack a unified standard for designating genes as “essential” or “nonessential” using CRISPRi. The ability of any CRISPRi system to identify essential genes depends on repression efficiency, the number of generations cells are grown after knockdown is induced, and the frequency at which CRISPRi suppressors exist within the cell population. High-efficiency systems (arbitrarily defined as >50-fold knockdown) are more likely to approximate null mutations for all queried genes, while low-efficiency or partially induced systems (<10-fold knockdown) will only report essentiality for a subset of genes that are most sensitive to knockdown (40); therefore, we recommend using high-efficiency systems for genome-wide analysis of essentiality using CRISPRi. For experiments using model bacteria with rigorously defined essential gene sets (e.g., E. coli [62] and B. subtilis [63]), the extent of CRISPRi knockdown and number of generations grown postknockdown can be calibrated to maximize recovery of essential genes (64). For nonmodel bacteria, such as Z. mobilis, we recommend comparing the viability or fitness of query genes to that of universally conserved essential genes (e.g., core ribosomal proteins). We further recommend removing repetitive sequences from CRISPRi constructs to reduce the likelihood that homologous recombination events inactivate CRISPRi and contribute to the assay background.

Our optimized CRISPRi system opens the door to high-throughput, systematic analysis of gene function in Z. mobilis. We envision that such screens will be invaluable for identifying genes involved in resistance to hydrolysate or biofuel inhibitors, genetic fingerprinting of hydrolysates from different plant sources or environments, and improving our understanding of the unique metabolism of Z. mobilis. We anticipate that this information will power the next generation of biofuel production strains, resulting in higher yields of advanced biofuels and bioproducts.

## MATERIALS AND METHODS

### Strains and growth conditions.

Strains are listed in Table 1. Escherichia coli was grown in Lennox LB broth (10 g tryptone, 5 g yeast extract, 5 g NaCl per liter; BD 240230) at 37°C aerobically in a flask with shaking at 250 rpm, in a culture tube on a roller drum, or in a deep 96-well plate with shaking at 900 rpm. Zymomonas mobilis was grown in DSMZ medium 10 (DSMZ10; 10 g peptone and 10 g yeast extract per liter plus 2% glucose) at 30°C aerobically without shaking. The medium was solidified with 1.5% agar for growth on plates. Antibiotics were added when necessary—for E. coli, 100 μg/ml ampicillin, 20 μg/ml chloramphenicol, or 30 μg/ml kanamycin, and for Z. mobilis, 100 μg/ml chloramphenicol, or 120 μg/ml kanamycin. Diaminopimelic acid (DAP) was added at 300 μM to support growth of *dap*-negative E. coli strains. IPTG (isopropyl β-d-1-thiogalactopyranoside) at 0.1 to 1 mM was added where indicated in the figures or figure legends. All strains were preserved in 15% glycerol at −80°C.

### Plasmid construction.

Plasmids and construction details are listed in Table 2, and a representative plasmid map is shown in Fig. S7 in the supplemental material; oligonucleotides and synthetic DNA are listed in Table 3. *pir*-dependent plasmids were propagated in E. coli strain BW25141 (sJMP146) and other plasmids in E. coli strain DH10B (sJMP032). Plasmids were assembled from fragments (linearized vector, PCR products, and/or synthetic DNA) using the NEBuilder hifi DNA assembly kit (catalog number E2621; New England Biolabs [NEB]). Plasmids were cut with restriction enzymes from NEB. Linearized plasmids were re-ligated using T4 DNA ligase (catalog number M0202; NEB). Fragments were amplified using Q5 DNA polymerase (catalog number M0491; NEB), followed by digestion with DpnI. Fragments were purified using the Monarch PCR & DNA cleanup kit (catalog number T1030; NEB) or the Zymo Research DNA Clean & Concentrator-5 kit (catalog number D4004) after digestion or amplification. Plasmids were transformed into electrocompetent E. coli cells using a Bio-Rad Gene Pulser Xcell on the EC1 setting. Plasmids were purified using the GeneJet plasmid miniprep kit (catalog number K0503; Thermo Scientific) or the PureLink HiPure Plasmid Midiprep kit (catalog number K210005; Invitrogen). Site-directed mutagenesis of plasmids was performed by DNA synthesis with 2.5 U PfuUltra II fusion HS DNA polymerase (Agilent), 0.2 μM single oligonucleotide bearing the change, 0.2 mM deoxynucleoside triphosphates (dNTPs), and 50 ng plasmid DNA in a 25-μl reaction mixture with a 1-min/kb extension time at 68°C, followed by DpnI digestion. sgRNA-encoding sequences were cloned into CRISPRi plasmids between the BsaI sites with inserts prepared by one of two methods. In method one, two 24-nucleotide (nt) oligonucleotides were designed to overlap such that when annealed, their ends would be complementary to the BsaI-cut ends on the vector. Oligonucleotides (2 μM each) were annealed in 1× CutSmart buffer (NEB) at 95°C for 5 min, followed by cooling to room temperature. For method two, fragments were amplified by PCR with primers oJMP197 and oJMP198 from a 78-nt oligonucleotide, followed by digestion with BsaI-HF-v2 (catalog number R3733; NEB) and purification with the Monarch DNA purification kit (NEB) following the manufacturer’s oligonucleotide purification protocol. Inserts (2 μl of a 1:40 dilution of annealed oligonucleotides or 2 ng purified digested PCR product) were ligated into 50 ng BsaI-digested vector. Oligonucleotides and synthetic DNA gBlocks were purchased from Integrated DNA Technologies (Coralville, IA). Sequencing was performed by Functional Biosciences (Madison, WI).

**TABLE 2:** Plasmids

Plasmid	Description^*a*^	Construction/notes	Marker(s)^*b*^	Reference or source
pJMP445	pRL814	Broad-host-range plasmid, pBBR1 *ori*, sfGFP	Spec^r^	67
pJMP1039	pTn7C1	Tn*7* transposase expression	Amp^r^	41
pJMP1183	pTn7C89.1	Mobile-CRISPRi RFP test plasmid (RR1 sgRNA)	Amp^r^, Kan^r^	41
pJMP1185	pTn7C90.1	Mobile-CRISPRi RFP test plasmid (nontargeting sgRNA)	Amp^r^, Kan^r^	41
pJMP1337	MCi (ICE::CRISPRi, *Spy* dCas9)		Amp^r^, Kan^r^	41
pJMP1339	MCi (Tn*7*::CRISPRi, *Hsa* dCas9)		Amp^r^, Kan^r^	41
pJMP1356	MCi (*Hsa* dCas9)		Amp^r^, Cm^r^	41
pJMP2030	pRL814 (sfGFP ΔXhoI)	Eliminate XhoI site from sfGFP gene in pRL814 (pJMP445) by site-directed mutagenesis with oJMP076	Spec^r^	This study
pJMP2044	MCi (Cm^r^) (*Spy* dCas9)	pJMP1356 cut with AscI and SpeI, assembled with *Spy* dCas9, amplified from pJMP1337 with oJMP072 and oJMP073	Amp^r^, Cm^r^	This study
pJMP2046	MCi-vA-GFP_BsaI	pJMP2044 cut with EcoRI and PmeI, assembled with gBlock oJMP079 and sfGFP (no XhoI), amplified from pJMP2030 with oJMP074 and oJMP075	Amp^r^, Cm^r^	This study
pJMP2048	MCi-vA-GFP_gfp	pJMP2044 cut with EcoRI and PmeI, assembled with gBlock oJMP080 and sfGFP (no XhoI), amplified from pJMP2030 with oJMP074 and oJMP075	Amp^r^, Cm^r^	This study
pJMP2093	MCi-vB-GFP_BsaI	pJMP2046 cut with EcoRI, assembled with gBlock oJMP191	Amp^r^, Cm^r^	This study
pJMP2095	MCi-vB-GFP_gfp	pJMP2048 cut with EcoRI, assembled with gBlock oJMP192	Amp^r^, Cm^r^	This study
pJMP2132	MCi-vB_BsaI	pJMP2093 cut with PmeI and re-ligated to remove sfGFP	Amp^r^, Cm^r^	This study
pJMP2316	pSRK-kan	Broad-host-range plasmid, pBBR1 *ori*	Kan^r^	68
pJMP2317	pSRK-kan-sgRNA	Vector backbone amplified from pJMP2316 with oJMP313 and oJMP314, assembled with sgRNA cassette, amplified from pJMP2095 with oJMP315 and oJMP316	Kan^r^	This study
pJMP2319	pSRK-kan-dCas9	Vector backbone amplified from pJMP2316 with oJMP313 and oJMP314, assembled with dCas9 cassette amplified from pJMP2095 with oJMP317 and oJMP318	Kan^r^	This study
pJMP2367	MCi-vC-GFP_BsaI	Assemble EcoRI-cut pJMP2093 with gBlock oJMP347	Amp^r^, Cm^r^	This study
pJMP2369	MCi-vD-GFP_BsaI	Assemble EcoRI-cut pJMP2093 with gBlock oJMP348	Amp^r^, Cm^r^	This study
pJMP2371	MCi-vE-GFP_BsaI	Assemble EcoRI-cut pJMP2093 with gBlock oJMP349	Amp^r^, Cm^r^	This study
pJMP2373	MCi-vF-GFP_BsaI	Assemble EcoRI-cut pJMP2093 with gBlock oJMP350	Amp^r^, Cm^r^	This study
pJMP2375	MCi-vC-GFP_gfp	Assemble EcoRI-cut pJMP2093 with gBlock oJMP351	Amp^r^, Cm^r^	This study
pJMP2377	MCi-vD-GFP_gfp	Assemble EcoRI-cut pJMP2093 with gBlock oJMP352	Amp^r^, Cm^r^	This study
pJMP2379	MCi-vE-GFP_gfp	Assemble EcoRI-cut pJMP2093 with gBlock oJMP353	Amp^r^, Cm^r^	This study
pJMP2381	MCi-vF-GFP_gfp	Assemble EcoRI-cut pJMP2093 with gBlock oJMP354	Amp^r^, Cm^r^	This study
pJMP2391	MCi-vB_*hpnC*-1 (ZMO869)	Annealed oJMP355 and oJMP356 ligated into BsaI-cut pJMP2132	Amp^r^, Cm^r^	This study
pJMP2393	MCi-vB_*hpnC*-2 (ZMO869)	Annealed oJMP357 and oJMP358 ligated into BsaI-cut pJMP2132	Amp^r^, Cm^r^	This study
pJMP2395	MCi-vB_*hpnF*-1 (ZMO872)	Annealed oJMP359 and oJMP360 ligated into BsaI-cut pJMP2132	Amp^r^, Cm^r^	This study
pJMP2397	MCi-vB_*hpnF*-2 (ZMO872)	Annealed oJMP361 and oJMP362 ligated into BsaI-cut pJMP2132	Amp^r^, Cm^r^	This study
pJMP2399	MCi-vB_*hpnH*-1 (ZMO874)	Annealed oJMP363 and oJMP364 ligated into BsaI-cut pJMP2132	Amp^r^, Cm^r^	This study
pJMP2401	MCi-vB_*hpnH*-2 (ZMO874)	Annealed oJMP365 and oJMP366 ligated into BsaI-cut pJMP2132	Amp^r^, Cm^r^	This study
pJMP2403	MCi-vB_*hpnI*-1 (ZMO972)	Annealed oJMP367 and oJMP368 ligated into BsaI-cut pJMP2132	Amp^r^, Cm^r^	This study
pJMP2405	MCi-vB_*hpnI*-2 (ZMO972)	Annealed oJMP369 and oJMP370 ligated into BsaI-cut pJMP2132	Amp^r^, Cm^r^	This study
pJMP2407	MCi-vB_*shc2*-1 (ZMO1548)	Annealed oJMP371 and oJMP372 ligated into BsaI-cut pJMP2132	Amp^r^, Cm^r^	This study
pJMP2409	MCi-vB_*shc2*-1 (ZMO1548)	Annealed oJMP373 and oJMP374 ligated into BsaI-cut pJMP2132	Amp^r^, Cm^r^	This study
pJMP2415	MCi-vB_rfp	Annealed oJMP003 and oJMP021 ligated into BsaI-cut pJMP2132	Amp^r^, Cm^r^	This study
pJMP2480	MCi-vC_BsaI	pJMP2367 cut with PmeI and re-ligated to remove sfGFP	Amp^r^, Cm^r^	This study
pJMP2509	MCi-vC-GFP_gmc1	Annealed oJMP400 and oJMP401 ligated into BsaI-cut pJMP2480	Amp^r^, Cm^r^	This study
pJMP2511	MCi-vC-GFP_gmc2	Annealed oJMP402 and oJMP403 ligated into BsaI-cut pJMP2480	Amp^r^, Cm^r^	This study
pJMP2513	MCi-vC-GFP_gmc3	Annealed oJMP404 and oJMP405 ligated into BsaI-cut pJMP2480	Amp^r^, Cm^r^	This study
pJMP2515	MCi-vC-GFP_gmc4	Annealed oJMP406 and oJMP407 ligated into BsaI-cut pJMP2480	Amp^r^, Cm^r^	This study
pJMP2517	MCi-vC-GFP_gmc5	Annealed oJMP408 and oJMP409 ligated into BsaI-cut pJMP2480	Amp^r^, Cm^r^	This study
pJMP2519	MCi-vC-GFP_gmc6	Annealed oJMP410 and oJMP411 ligated into BsaI-cut pJMP2480	Amp^r^, Cm^r^	This study
pJMP2521	MCi-vC-GFP_gmc7	Annealed oJMP412 and oJMP413 ligated into BsaI-cut pJMP2480	Amp^r^, Cm^r^	This study
pJMP2523	MCi-vC-GFP_gmc8	Annealed oJMP414 and oJMP415 ligated into BsaI-cut pJMP2480	Amp^r^, Cm^r^	This study
pJMP2525	MCi-vC-GFP_gmc9	Annealed oJMP416 and oJMP417 ligated into BsaI-cut pJMP2480	Amp^r^, Cm^r^	This study
pJMP2597	MCi-vC-*pdc*-1 (ZMO1360)	Annealed oJMP467 and oJMP468 ligated into BsaI-cut pJMP2480	Amp^r^, Cm^r^	This study
pJMP2598	MCi-vC-*pdc*-3 (ZMO1360)	Annealed oJMP469 and oJMP470 ligated into BsaI-cut pJMP2480	Amp^r^, Cm^r^	This study
pJMP2599	MCi-vC-*rplL* (ZMO0728)	Amplify from opZ1-1-13 with oJMP197 and oJMP198, digest fragment with BsaI, and ligate into BsaI-cut pJMP2480	Amp^r^, Cm^r^	This study

a MCi, Mobile-CRISPRi; ICE, integrative conjugative element; *Hsa*, *Homo sapiens* codon-optimized dCas9; vA to vF, sgRNA promoter variants; RFP, red fluorescent protein; GFP, green fluorescent protein; BsaI, vector with BsaI cloning site for sgRNA.

b Cm^r^, chloramphenicol resistance; Amp^r^, ampicillin resistance, Kan^r^, kanamycin resistance.

**TABLE 3:** Oligonucleotides and synthetic DNA

Oligonucleotide	Sequence (5′→3′)	Description	Usage
oJMP003	TAGTAACTTTCAGTTTAGCGGTCT	rfp_T	pJMP2415
oJMP021	AAACAGACCGCTAAACTGAAAGTT	rfp_B	pJMP2415
oJMP057	CCAAGGTGCATCCTCTCATT	Zmo_Tn7_check_A	
oJMP058	TATCGGACAATCGGGAAGAC	Zmo_Tn7_check_B	
oJMP059	GCCCCGATCGTCTATGCTAT	Zmo_Tn7_check_C	
oJMP060	CGCCCCTCTTTAATACGACG	Tn7R_check	
oJMP072	CGCTTTTTTTACGTCTGCAGACTAGTAAAATTTATCAAAAAGAGTGTTGACTTGTGAGCGGATAACAATGATACTTAGATTCAATTGTGAGCGGATAACAATTGAGCGAGAAGGAGGACTAGTATGGATAAGAAATACTCAATAGGC	T7A1_O3O4-dcas9_F	pJMP2044
oJMP073	TTTGGTACCGAGGCTGCAA	T7A1_O3O4-dcas9_R	pJMP2044
oJMP074	GGAGAAGAACTTTTCACTGGAGT	sfgfp_F	pJMP2046, pJMP2048
oJMP075	GCAAATCCAGGAGGTCGTTTAAACTTATTATTTGTAGAGCTCATCCATGCCATGTG	sfgfp_R	pJMP2046, pJMP2048
oJMP076	ATGGAAACATTCTTGGACACAAACTGGAGTACAACTTTAACTCACACAATG	sfgfp_no_XhoI_QC	pJMP2030
oJMP079	ACCTATCGACTGAGCTGAAAGAATTCGCTCACTCATTAGGCACCCCAGGCTTTACACTTTATGCTTCCGGCTCGTATAATGTCTAGTTGAGACCAACTTTGGTCTCCACCATAGCGGTCGGTCTCTGTTTAAGAGCTATGCTGGAAACAGCATAGCAAGTTTAAATAAGGCTAGTCCGTTATCAACTTGAAAAAGTGGCACCGAGTCGGTGCTTTTTTTTTGAATTCATGTGGCTGACCGTTCTGTTGTCTCTCGCTCTTCCGAGTAGACGAACAATAAGGCCTCCCTAACGGGGGGCCTTTTTTATTGATAACAAAAGTCAGTGCTTCCGCTATTTCCAAAATACCGGGCTAATACGGTTTAAACGAAAATTTATCAAAAAGAGTATTGACTTAAAGTCTAACCTATAGGATACTTACAGCCAGATCTGAGCGAGAAGGAGGTAAAGTATGAGCAAAGGAGAAGAACTTTTCACTGG	Zmo_M-Ci_gBlock_BsaI	pJMP2046
oJMP080	ACCTATCGACTGAGCTGAAAGAATTCGCTCACTCATTAGGCACCCCAGGCTTTACACTTTATGCTTCCGGCTCGTATAATGTCTAGTCATCTAATTCAACAAGAATTGTTTAAGAGCTATGCTGGAAACAGCATAGCAAGTTTAAATAAGGCTAGTCCGTTATCAACTTGAAAAAGTGGCACCGAGTCGGTGCTTTTTTTTTGAATTCATGTGGCTGACCGTTCTGTTGTCTCTCGCTCTTCCGAGTAGACGAACAATAAGGCCTCCCTAACGGGGGGCCTTTTTTATTGATAACAAAAGTCAGTGCTTCCGCTATTTCCAAAATACCGGGCTAATACGGTTTAAACGAAAATTTATCAAAAAGAGTATTGACTTAAAGTCTAACCTATAGGATACTTACAGCCAGATCTGAGCGAGAAGGAGGTAAAGTATGAGCAAAGGAGAAGAACTTTTCACTGG	Zmo_M-Ci_gBlock_GR1	pJMP2048
oJMP191	ACCTATCGACTGAGCTGAAAGAATTCGCTCACTCATTAGGCACCCCAGGCTTTACAATTGTGAGCGCTCACAATTATAATGTCTAGTTGAGACCAACTTTGGTCTCCACCATAGCGGTCGGTCTCTGTTTAAGAGCTATGCTGGAAACAGCATAGCAAGTTTAAATAAGGCTAGTCCGTTATCAACTTGAAAAAGTGGCACCGAGTCGGTGCTTTTTTTTTGAATTCATGTGGCTGACCGTTCTGTT	BsaI_gBlock	pJMP2093
oJMP192	ACCTATCGACTGAGCTGAAAGAATTCGCTCACTCATTAGGCACCCCAGGCTTTACAATTGTGAGCGCTCACAATTATAATGTCTAGTCATCTAATTCAACAAGAATTGTTTAAGAGCTATGCTGGAAACAGCATAGCAAGTTTAAATAAGGCTAGTCCGTTATCAACTTGAAAAAGTGGCACCGAGTCGGTGCTTTTTTTTTGAATTCATGTGGCTGACCGTTCTGTT	GR1_gblock	pJMP2095
oJMP197	GTCTATTGAAGTACCTGC	Z1_amplify_A	pJMP2599
oJMP198	CGATGCTCCTCCAAGATA	Z1_amplify_B	pJMP2599
oJMP313	GCTAGCAATTCGAAAGCAAATTCGACCC	pSRK-V-F	pJMP2317, pJMP2319
oJMP314	ATTGCGTTGCGCTCACTGCC	pSRK-V-R	pJMP2317, pJMP2319
oJMP315	GGCAGTGAGCGCAACGCAATACCTATCGACTGAGCTGAAAGAAT	sgRNA-F	pJMP2317
oJMP316	GGTCGAATTTGCTTTCGAATTGCTAGCAACAGAACGGTCAGCCACAT	sgRNA-R	pJMP2317
oJMP317	GGCAGTGAGCGCAACGCAATTGCAGACTAGTAAAATTTATCAAAAAGAGTGTTGAC	dCas9-F	pJMP2319
oJMP318	GGTCGAATTTGCTTTCGAATTGCTAGCGGCGCGCCTTATTACAGATCTTC	dCas9-R	pJMP2319
oJMP347	ACCTATCGACTGAGCTGAAAGAATTCGGAAAATTTTTTTTCAAAAGTACTTGAAATTGTGAGCGCTCACAATTATAATTCTAGTAGAGACCAACTTTGGTCTCCACCATAGCGGTCGGTCTCTGTTTAAGAGCTATGCTGGAAACAGCATAGCAAGTTTAAATAAGGCTAGTCCGTTATCAACTTGAAAAAGTGGCACCGAGTCGGTGCTTTTTTTTTGAATTCATGTGGCTGACCGTTCTGTT	C_gBlock	pJMP2367
oJMP348	ACCTATCGACTGAGCTGAAAGAATTCGGAAAATTTTTTTTCAAAAGTACTTGAATTGTGAGCGGATAACAATTATAATTCTAGTAGAGACCAACTTTGGTCTCCACCATAGCGGTCGGTCTCTGTTTAAGAGCTATGCTGGAAACAGCATAGCAAGTTTAAATAAGGCTAGTCCGTTATCAACTTGAAAAAGTGGCACCGAGTCGGTGCTTTTTTTTTGAATTCATGTGGCTGACCGTTCTGTT	D_gBlock	pJMP2369
oJMP349	ACCTATCGACTGAGCTGAAAGAATTCGGAAAATTTTTTTTCAAAAGTACTTTACAATTGTGAGCGCTCACAATTATAATTCTAGTAGAGACCAACTTTGGTCTCCACCATAGCGGTCGGTCTCTGTTTAAGAGCTATGCTGGAAACAGCATAGCAAGTTTAAATAAGGCTAGTCCGTTATCAACTTGAAAAAGTGGCACCGAGTCGGTGCTTTTTTTTTGAATTCATGTGGCTGACCGTTCTGTT	E_gBlock	pJMP2371
oJMP350	ACCTATCGACTGAGCTGAAAGAATTCGGAAAATTTTTTTTCAAAAGTACTTTAAATTGTGAGCGGATAACAATTATAATTCTAGTAGAGACCAACTTTGGTCTCCACCATAGCGGTCGGTCTCTGTTTAAGAGCTATGCTGGAAACAGCATAGCAAGTTTAAATAAGGCTAGTCCGTTATCAACTTGAAAAAGTGGCACCGAGTCGGTGCTTTTTTTTTGAATTCATGTGGCTGACCGTTCTGTT	F_gBlock	pJMP2373
oJMP351	ACCTATCGACTGAGCTGAAAGAATTCGGAAAATTTTTTTTCAAAAGTACTTGAAATTGTGAGCGCTCACAATTATAATTCTAGTCATCTAATTCAACAAGAATTGTTTAAGAGCTATGCTGGAAACAGCATAGCAAGTTTAAATAAGGCTAGTCCGTTATCAACTTGAAAAAGTGGCACCGAGTCGGTGCTTTTTTTTTGAATTCATGTGGCTGACCGTTCTGTT	C-gfp_gBlock	pJMP2375
oJMP352	ACCTATCGACTGAGCTGAAAGAATTCGGAAAATTTTTTTTCAAAAGTACTTGAATTGTGAGCGGATAACAATTATAATTCTAGTCATCTAATTCAACAAGAATTGTTTAAGAGCTATGCTGGAAACAGCATAGCAAGTTTAAATAAGGCTAGTCCGTTATCAACTTGAAAAAGTGGCACCGAGTCGGTGCTTTTTTTTTGAATTCATGTGGCTGACCGTTCTGTT	D-gfp_gBlock	pJMP2377
oJMP353	ACCTATCGACTGAGCTGAAAGAATTCGGAAAATTTTTTTTCAAAAGTACTTTACAATTGTGAGCGCTCACAATTATAATTCTAGTCATCTAATTCAACAAGAATTGTTTAAGAGCTATGCTGGAAACAGCATAGCAAGTTTAAATAAGGCTAGTCCGTTATCAACTTGAAAAAGTGGCACCGAGTCGGTGCTTTTTTTTTGAATTCATGTGGCTGACCGTTCTGTT	E-gfp_gBlock	pJMP2379
oJMP354	ACCTATCGACTGAGCTGAAAGAATTCGGAAAATTTTTTTTCAAAAGTACTTTAAATTGTGAGCGGATAACAATTATAATTCTAGTCATCTAATTCAACAAGAATTGTTTAAGAGCTATGCTGGAAACAGCATAGCAAGTTTAAATAAGGCTAGTCCGTTATCAACTTGAAAAAGTGGCACCGAGTCGGTGCTTTTTTTTTGAATTCATGTGGCTGACCGTTCTGTT	F-gfp_gBlock	pJMP2381
oJMP355	TAGTTCCTTTTCCAGAAACCAAAG	zmo_hpnC_A_T	pJMP2391
oJMP356	AAACCTTTGGTTTCTGGAAAAGGA	zmo_hpnC_A_B	pJMP2391
oJMP357	TAGTATAATAATAGGCCGATATTC	zmo_hpnC_B_T	pJMP2393
oJMP358	AAACGAATATCGGCCTATTATTAT	zmo_hpnC_B_B	pJMP2393
oJMP359	TAGTCGGGCTATGATGAAAAGCCG	zmo_hpnF_A_T	pJMP2395
oJMP360	AAACCGGCTTTTCATCATAGCCCG	zmo_hpnF_A_B	pJMP2395
oJMP361	TAGTCGGGTGGCCTTTTGGATAAT	zmo_hpnF_B_T	pJMP2397
oJMP362	AAACATTATCCAAAAGGCCACCCG	zmo_hpnF_B_B	pJMP2397
oJMP363	TAGTATCCGTAAAACCTGACTAAA	zmo_hpnH_A_T	pJMP2399
oJMP364	AAACTTTAGTCAGGTTTTACGGAT	zmo_hpnH_A_B	pJMP2399
oJMP365	TAGTGGTTCAAGCATCAAAACCAG	zmo_hpnH_B_T	pJMP2401
oJMP366	AAACCTGGTTTTGATGCTTGAACC	zmo_hpnH_B_B	pJMP2401
oJMP367	TAGTTGTCAAAAGGACATGCAGGA	zmo_hpnI_A_T	pJMP2403
oJMP368	AAACTCCTGCATGTCCTTTTGACA	zmo_hpnI_A_B	pJMP2403
oJMP369	TAGTCCACCACAGCACGACAATCG	zmo_hpnI_B_T	pJMP2405
oJMP370	AAACCGATTGTCGTGCTGTGGTGG	zmo_hpnI_B_B	pJMP2405
oJMP371	TAGTATCGGAAGCCGGTTTATACG	zmo_shc2_A_T	pJMP2407
oJMP372	AAACCGTATAAACCGGCTTCCGAT	zmo_shc2_A_B	pJMP2407
oJMP373	TAGTTTGCCGTCGGTTCAGGCCGC	zmo_shc2_B_T	pJMP2409
oJMP374	AAACGCGGCCTGAACCGACGGCAA	zmo_shc2_B_B	pJMP2409
oJMP400	TAGTTTCCGTTGGGATCTTTCGAA	gmc1_T	pJMP2409
oJMP401	AAACTTCGAAAGATCCCAACGGAA	gmc1_B	pJMP2409
oJMP402	TAGTTAGTACATAACCTTCGGGCA	gmc2_T	pJMP2411
oJMP403	AAACTGCCCGAAGGTTATGTACTA	gmc2_B	pJMP2411
oJMP404	TAGTGTCAGAGTAGTGTCAAGTGT	gmc3_T	pJMP2413
oJMP405	AAACACACTTGACACTACTCTGAC	gmc3_B	pJMP2413
oJMP406	TAGTGGCAAAGCATTGAAAACCAT	gmc4_T	pJMP2415
oJMP407	AAACATGGTTTTCAATGCTTTGCC	gmc4_B	pJMP2415
oJMP408	TAGTGCGTTCCTGTACATAACCCT	gmc5_T	pJMP2417
oJMP409	AAACAGGGTTATGTACAGGAACGC	gmc5_B	pJMP2417
oJMP410	TAGTCATCTAATTCAACAAGAATT	gmc6_T	pJMP2419
oJMP411	AAACAATTCTTGTTGAATTAGATG	gmc6_B	pJMP2419
oJMP412	TAGTATGTTGTCACGCTTTTCGTT	gmc7_T	pJMP2421
oJMP413	AAACAACGAAAAGCGTGACAACAT	gmc7_B	pJMP2421
oJMP414	TAGTAGTAGTGCAAAGAAATTTAA	gmc8_T	pJMP2423
oJMP415	AAACTTAAATTTCTTTGCACTACT	gmc8_B	pJMP2423
oJMP416	TAGTAAACGACAGATTGTGTCGAC	gmc9_T	pJMP2425
oJMP417	AAACGTCGACACAATCTGTCGTTT	gmc9_B	pJMP2425
oJMP467	TAGTGACAAGCCGCTCCGCTAAAT	zmo1360-pdc-1-T	pJMP2597
oJMP468	AAACATTTAGCGGAGCGGCTTGTC	zmo1360-pdc-1-B	pJMP2597
oJMP469	TAGTGACGGCTGCTGCTGCGCCTT	zmo1360-pdc-3-T	pJMP2598
oJMP470	AAACAAGGCGCAGCAGCAGCCGTC	zmo1360-pdc-3-B	pJMP2598
opZ1-1-13	GTCTATTGAAGTACCTGCGGTCTCTTAGTAAGTTCAGCTGCTTCAAGAAGTTTAGAGACCTATCTTGGAGGAGCATCG	opZ1-1-13-zmo0728_rplL	pJMP2599

### Transfer of CRISPRi system to E. coli and Z. mobilis.

Strains with a chromosomally located CRISPRi expression cassette were constructed by triparental mating of two donor strains—one with a plasmid encoding Tn*7* transposase and another with a plasmid containing a Tn*7* transposon encoding the CRISPRi system—and a recipient strain (either E. coli BW25113 or Z. mobilis ZM4 [PK15436]). The method was as described in Peters et al. (41), with several modifications. Briefly, all matings used E. coli WM6026, which is *pir^+^* to support *pir*-dependent plasmid replication, *dap* negative, making it dependent on diaminopimelic acid (DAP) for growth, and encodes the RP4 transfer machinery required for conjugation. Donor strains were grown for ∼16 h at 37°C in LB plus 100 μg/ml ampicillin and 300 μM DAP. The E. coli recipient was grown for ∼16 h at 37°C in LB. The Z. mobilis recipient was grown for ∼24 to 30 h at 30°C in DSMZ10. Cells were centrifuged at 4,000 × *g* for 5 min and gently resuspended twice in an equal volume of fresh medium with no antibiotic or DAP. For E. coli recipients, 700 μl LB and 100 μl each donor and recipient were mixed in a sterile 1.5-ml microcentrifuge tube. For Z. mobilis recipients, 200 μl DSMZ10, 500 μl Z. mobilis, 200 μl transposase donor, and 100 μl transposon donor were mixed in a sterile 1.5-ml microcentrifuge tube. Cells were centrifuged at 4,000 × *g* for 3 min, gently resuspended in 25 μl LB or DSMZ10, and pipetted onto a 13-mm cellulose filter (number HAWG01300; MF-Millipore) placed on a prewarmed agar plate (LB for E. coli or DSMZ10 for Z. mobilis). Plates were incubated at 37°C for 2 to 6 h for E. coli and 30°C for 24 h for Z. mobilis. After the incubation period, using sterile forceps, filters were placed into sterile 1.5-ml microcentrifuge tubes containing 200 μl sterile 1× phosphate-buffered saline (PBS), vortexed for 20 s to dislodge cells from filters, diluted in 1× PBS, and plated on appropriate medium for the recipient, with antibiotic to select for the transposon (see above) and no DAP (to select against the donor). The efficiency of transposition was generally ∼1 in 10^3^ for E. coli and ∼1 in 10^5^ to 10^6^ for Z. mobilis. Isolated colonies were generally obtained from ∼10 to 100 μl of a 1:100 dilution per plate, and isolated colonies were restruck for isolation to ensure purity.

### CRISPRi insertion onto Z. mobilis chromosome.

Insertion of the CRISPRi expression cassette into the Tn*7 att* site downstream from *glmS* in Z. mobilis was confirmed by PCR with primers oJMP057 and oJMP058 (flanking the insertion site) and oJMP059 and oJMP060 (upstream from the insertion site and within the CRISPRi transposon).

### CRISPRi stability in Z. mobilis.

Z. mobilis strains with chromosomally located CRISPRi expression cassettes (6 individual isolates) were grown in liquid culture medium with antibiotic selection to saturation. This culture was serially diluted to 10^−5^ into nonselective medium (starting OD_600_ of ∼0.00002) and grown for ∼17 generations back to saturation (OD_600_ of ∼2.0). Dilution and growth were repeated 2 additional times for a total of ∼50 generations prior to plating on nonselective plates. Forty-eight isolated colonies were selected and patched on selective and nonselective plates, and all strains retained the ability to grow on the antibiotic to which resistance was conferred by the chromosomally located CRISPRi expression cassette.

### GFP and RFP knockdown assays.

GFP or RFP knockdown was measured using a plate reader (Tecan Infinite 200 Pro M Plex). Cell density was determined by OD_600_, and fluorescence was measured by excitation/emission at 482/515 nm for GFP and 584/607 nm for RFP. Initial cultures (*n* = 4) were grown from single colonies to saturation (∼30 h for Z. mobilis or ∼16 h for E. coli) in 1 ml medium in deep 96-well plates. These cultures were serially diluted 1:1,000 (Z. mobilis) or 1:10,000 (E. coli) into 1 ml fresh medium (no antibiotic and 0 to 1 mM IPTG as indicated) and grown back to saturation (∼24 to 30 h for Z. mobilis or ∼8 to 16 h for E. coli). Pelleted cells were resuspended in 1 ml 1× PBS and, if necessary, diluted, and 200 μl was transferred to a clear-bottom black microtiter plate and measured in the plate reader as indicated above. Fluorescence values were normalized to cell density and to measurements from strains not expressing GFP.

### Gene knockdown spot dilution assay.

Z. mobilis strains with chromosomally located CRISPRi expression cassettes (2 individual isolates) were grown to saturation in liquid culture medium with antibiotic selection. These cultures were serially diluted 1:10 in nonselective medium, and 3-μl amounts were spotted onto plates containing 0, 0.1 mM, or 1 mM IPTG that were incubated at 30°C aerobically prior to analysis.

### Gene knockdown growth assay.

Z. mobilis strains with chromosomally located CRISPRi expression cassettes (*n* = 2 individual isolates) were grown to saturation in liquid culture medium with antibiotic selection. These cultures were serially diluted 1:1,000 into nonselective medium with 0 or 0.1 mM IPTG and 0, 0.63%, 1.25%, or 2.5% isobutanol in a deep 96-well plate and incubated at 30°C aerobically prior to analysis of growth (measured as OD_600_).

### Data availability.

Plasmids and their sequences are available from Addgene (Addgene identification numbers 160073 to 160080).

## Supplementary Material

Supplemental file 1
