# Supplementary material for: A High-Efficacy CRISPR Interference System for Gene Function Discovery in Zymomonas mobilis
Source: Appl Environ Microbiol. 2020 Nov 10;86(23):e01621-20. doi: 10.1128/AEM.01621-20 (PMC7657623; doi:10.1128/AEM.01621-20)
Supplement: Supplemental file 1 [file AEM.01621-20-s0001.pdf]

Fig S1

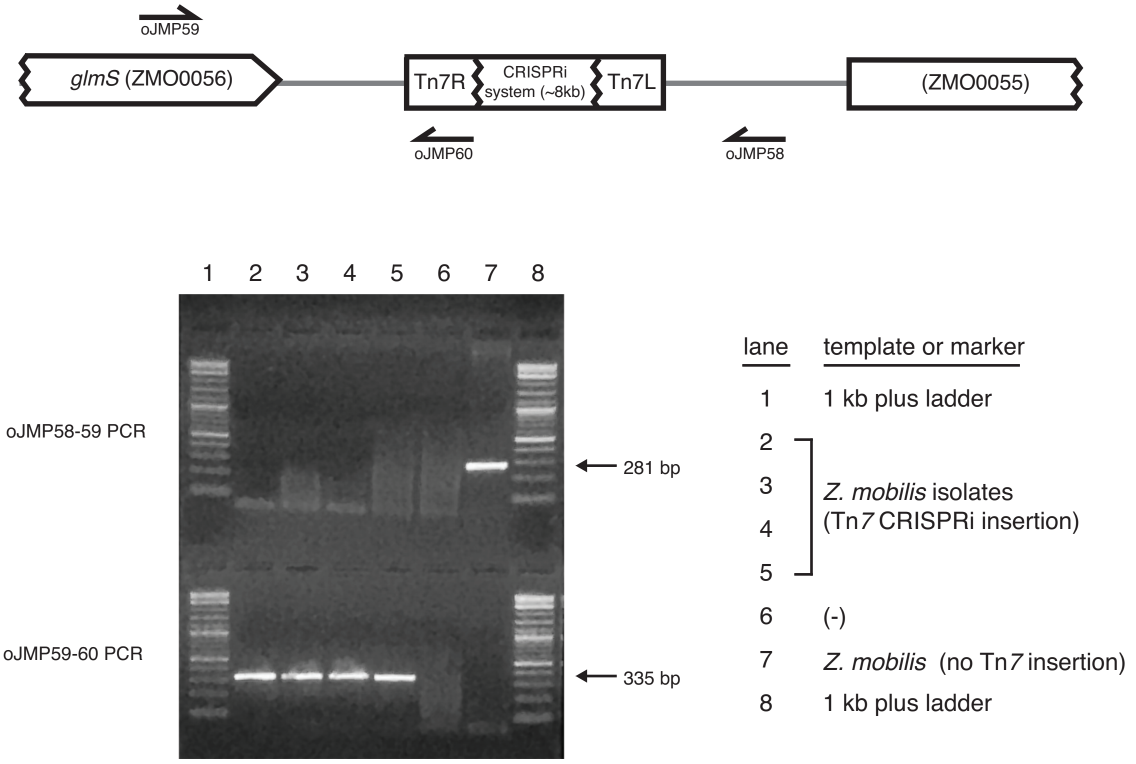

Fig S1 PCR test to confirm correct insertion of Mobile-CRISPRi into the *Z. mobilis* genome downstream of *glmS*.

Fig S2

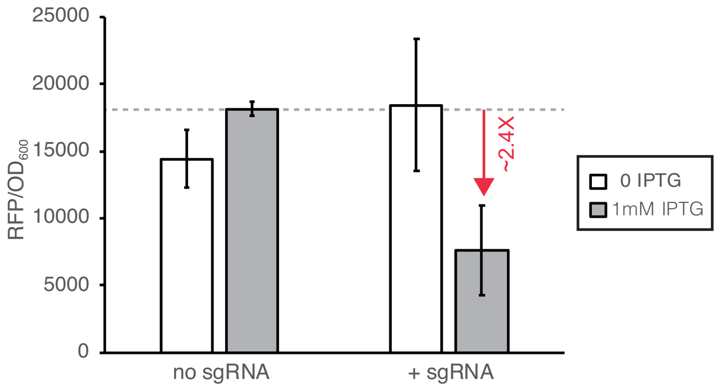

Fig S2 Knockdown of *mRFP* using the Mobile-CRISPRi system prior to optimization for *Z. mobilis*. Red arrow indicates fold change compared to control. Growth was measured by absorbance at 600nm.

Fig S3

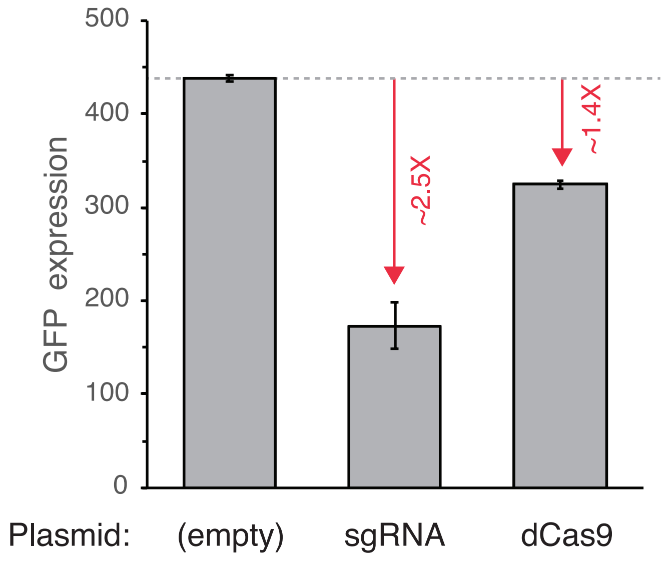

Fig S3 sgRNA is limiting in *Z. mobilis* promoter variant B CRISPRi system. Knockdown of GFP in *Z. mobilis* with the promoter variant B Mobile CRISPRi system on the chromosome and either additional sgRNA or additional dCas9 expressed from the broad host range plasmid pSRK-kan. Red arrows indicate fold change compared to control.

Fig S4

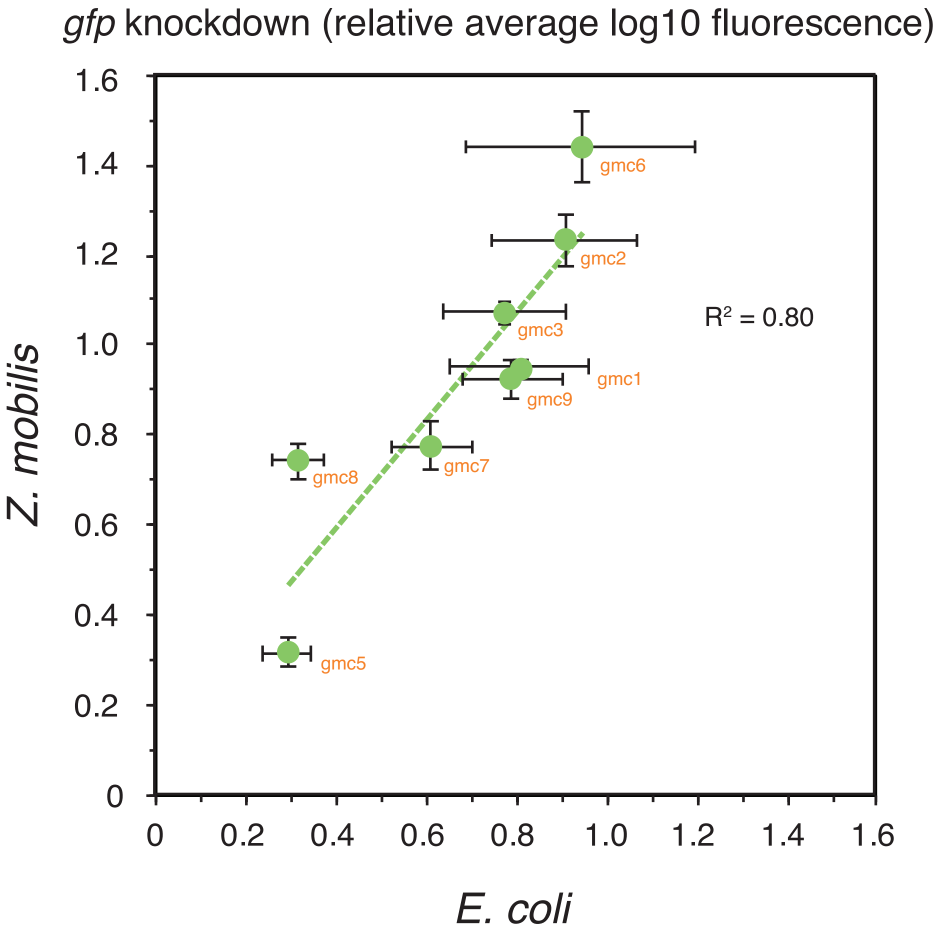

Fig S4 Comparison of *gfp* knockdown by mismatch sgRNAs in *Z. mobilis* vs *E. coli*. Graph shows *gfp* knockdown (relative average log10 fluorescence) of *Z. mobilis* vs *E. coli* CRISPRi strains expressing sgRNAs targeting *gfp* (indicated in orange). Linear trendline is dashed green.

FigS5 ***Zymomonas mobilis* Hopanoid Synthesis Pathway (simplified)**

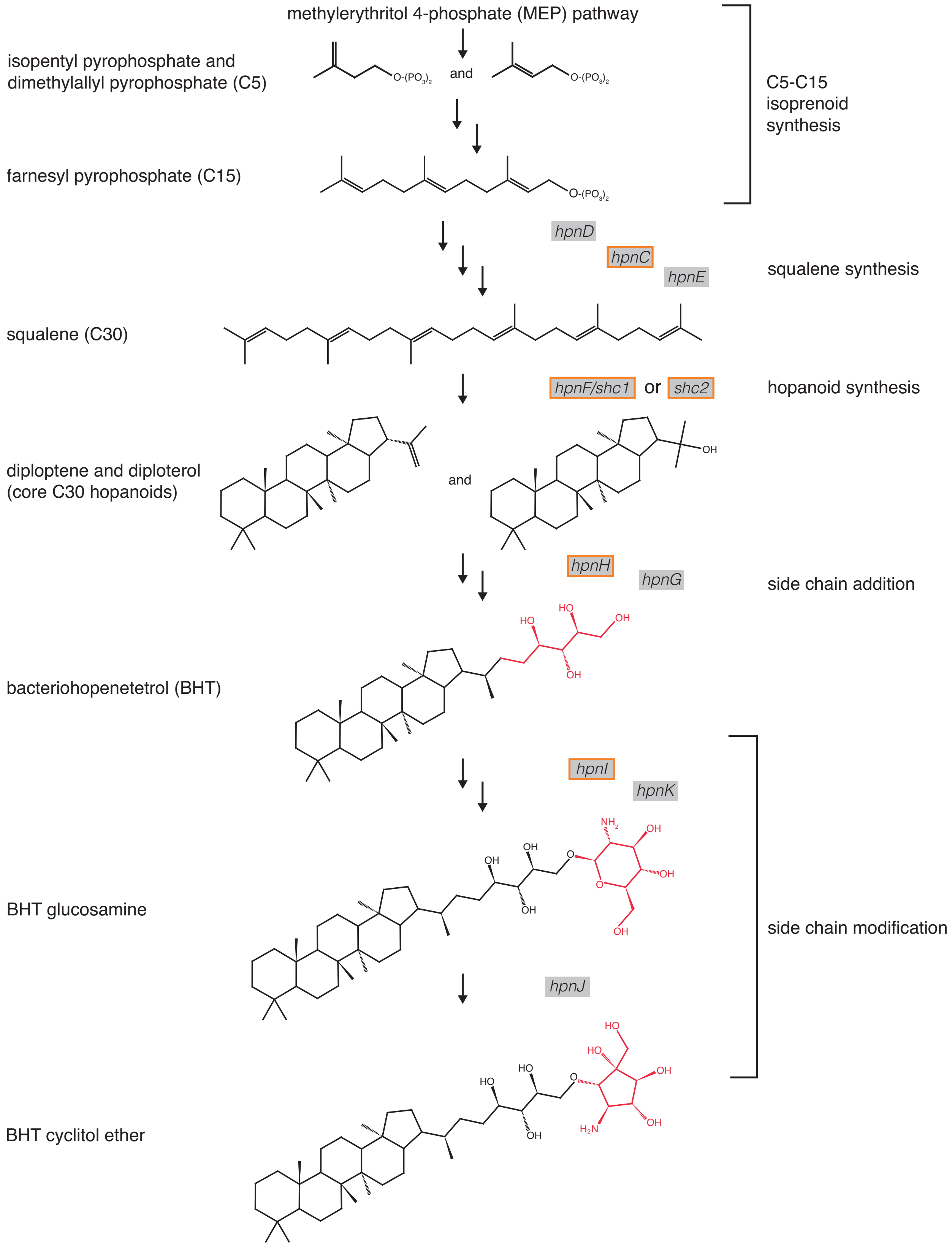

Fig S5 Hopanoid lipid synthesis pathway in *Z. mobilis*. Relevant lipids and their structures are shown (some intermediates omitted for simplicity). Genes encoding enzymes are indicated in grey boxes next to the arrows, with CRISPRi target genes boxed in orange. Red is used to highlight the change from the previous structure.

Fig S6

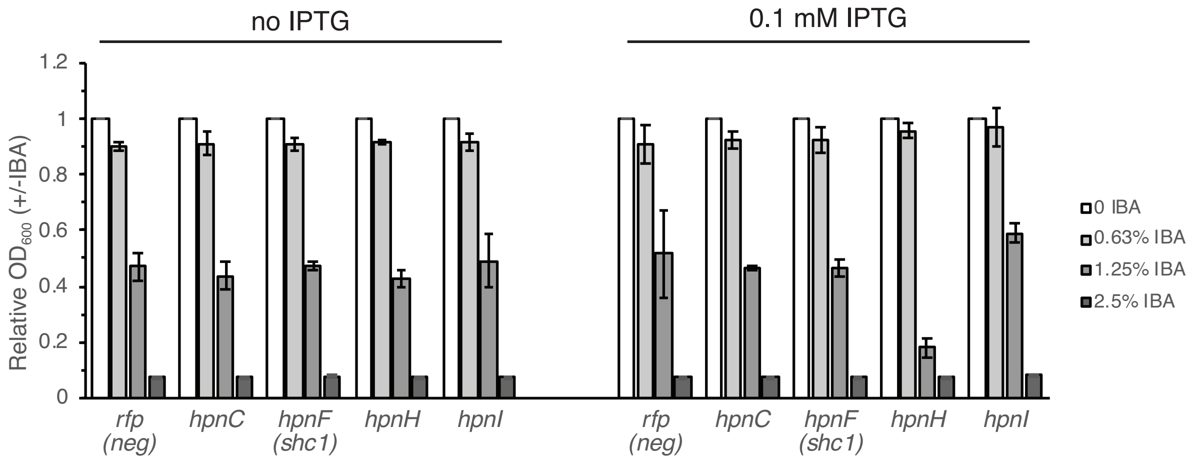

Fig S6 CRISPRi knockdown of hopanoid lipid synthesis related genes. Expanded version of data in Fig 4C showing additional concentrations of isobutanol (0.63%, 1.25%, 2.5% and 0 or 0.1 mM IPTG. Standard deviation between 4 replicates is shown.

FigS7

A

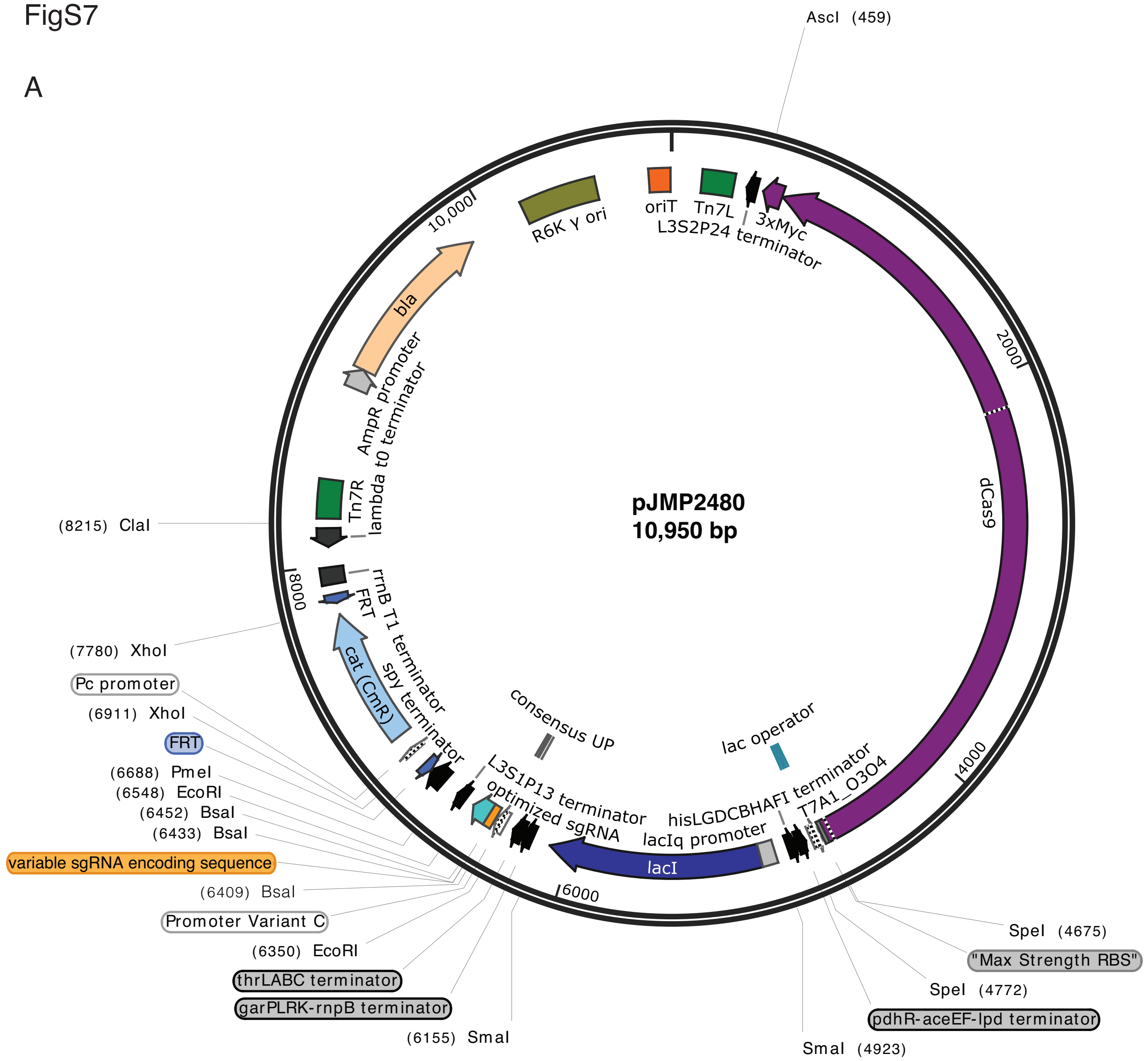

pJMP2480
10,950 bp
AscI (459)
oriT
Tn7L
L3S2P24 terminator
3xMyc
R6K γ ori
bla
AmpR promoter
t0 terminator
lambda t0 terminator
Tn7R
(8215) ClaI
rrnB T1 terminator
FRT
cat (CmR)
spy terminator
(7780) XhoI
Pc promoter
(6911) XhoI
FRT
(6688) PmeI
(6548) EcoRI
(6452) BsaI
(6433) BsaI
variable sgRNA encoding sequence
(6409) BsaI
Promoter Variant C
(6350) EcoRI
thrLABC terminator
garPLRK-rnpB terminator
(6155) SmaI
consensus UP
L3S1P13 terminator
optimized sgRNA
lac operator
lacI
lacIq promoter
hisLGDCBHAFI terminator
T7A1_O3O4
dCas9
SpeI (4675)
"Max Strength RBS"
SpeI (4772)
pdhR-aceEF-lpd terminator
SmaI (4923)
10,000
2000
4000
6000
8000

B

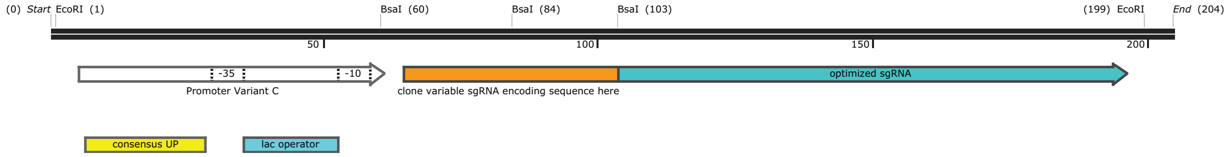

(0) Start EcoRI (1)
BsaI (60)
BsaI (84)
BsaI (103)
(199) EcoRI
End (204)
50
100
150
200
-35
-10
Promoter Variant C
clone variable sgRNA encoding sequence here
optimized sgRNA
consensus UP
lac operator

C

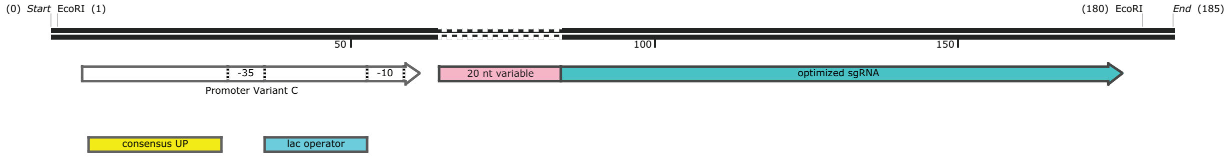

(0) Start EcoRI (1)
(180) EcoRI
End (185)
50
100
150
-35
-10
Promoter Variant C
20 nt variable
optimized sgRNA
consensus UP
lac operator

Fig S7 Mobile CRISPRi system for *Zymomonas mobilis*. (A) Plasmid map of pJMP2480 showing CRISPRi system located on a transposon (between Tn*7*L and Tn*7*R in dark green) that can be transferred to *Z. mobilis* genome via Tn*7* transposase-mediated transposition. The modular design enables parts to be exchanged as necessary by restriction enzyme digest (restriction enzyme sites shown) followed by Gibson assembly. All components are separated by transcriptional terminators. Once integrated into the genome, the system is stable without antibiotic selection for > 50 generations. The *cat* antibiotic cassette (light blue) is flanked by FRT sites (dark blue) to enable removal from the genome using Flp-recombinase, if desired. Full sequence of the plasmid is available at addgene.org. (B) Zoomed in view of the sgRNA expression cassette (between EcoRI sites). The 20 nt variable region of the sgRNA can be cloned between the BsaI sites. (C) Zoomed in view of the sgRNA expression cassette showing location of the 20 nt variable region (pink).
